# Community Engagement in the Development of an mHealth-Enabled Physical Activity and Cardiovascular Health Intervention (Step It Up): Pilot Focus Group Study

**DOI:** 10.2196/10944

**Published:** 2019-01-04

**Authors:** Joniqua Nashae Ceasar, Sophie Elizabeth Claudel, Marcus R Andrews, Kosuke Tamura, Valerie Mitchell, Alyssa T Brooks, Tonya Dodge, Sherine El-Toukhy, Nicole Farmer, Kimberly Middleton, Melanie Sabado-Liwag, Melissa Troncoso, Gwenyth R Wallen, Tiffany M Powell-Wiley

**Affiliations:** 1 National Heart, Lung, and Blood Institute National Institutes of Health Bethesda, MD United States; 2 Clinical Center National Institutes of Health Bethesda, MD United States; 3 Department of Psychology George Washington University Washington, DC United States; 4 Department of Public Health California State University Los Angeles, CA United States; 5 Uniformed Services University of the Health Sciences Bethesda, MD United States

**Keywords:** cardiovascular health, community-based participatory research, health behaviors, mHealth, mobile phone, physical activity, qualitative data

## Abstract

**Background:**

Community-based participatory research is an effective tool for improving health outcomes in minority communities. Few community-based participatory research studies have evaluated methods of optimizing smartphone apps for health technology-enabled interventions in African Americans.

**Objective:**

This study aimed to utilize focus groups (FGs) for gathering qualitative data to inform the development of an app that promotes physical activity (PA) among African American women in Washington, DC.

**Methods:**

We recruited a convenience sample of African American women (N=16, age range 51-74 years) from regions of Washington, DC metropolitan area with the highest burden of cardiovascular disease. Participants used an app created by the research team, which provided motivational messages through app push notifications and educational content to promote PA. Subsequently, participants engaged in semistructured FG interviews led by moderators who asked open-ended questions about participants’ experiences of using the app. FGs were audiorecorded and transcribed verbatim, with subsequent behavioral theory-driven thematic analysis. Key themes based on the Health Belief Model and emerging themes were identified from the transcripts. Three independent reviewers iteratively coded the transcripts until consensus was reached. Then, the final codebook was approved by a qualitative research expert.

**Results:**

In this study, 10 main themes emerged. Participants emphasized the need to improve the app by optimizing automation, increasing relatability (eg, photos that reflect target demographic), increasing educational material (eg, health information), and connecting with community resources (eg, cooking classes and exercise groups).

**Conclusions:**

Involving target users in the development of a culturally sensitive PA app is an essential step for creating an app that has a higher likelihood of acceptance and use in a technology-enabled intervention. This may decrease health disparities in cardiovascular diseases by more effectively increasing PA in a minority population.

## Introduction

Cardiovascular disease remains the leading cause of death in the United States, and African Americans bear a disproportionate burden, leading to significant and excessive morbidity and mortality [1,2]. Cardiovascular disease risk factors are more prevalent among both low-income and racial or ethnic minority populations, emphasizing the need to design interventions that address these risk factors and, ultimately, reduce health disparities [1]. A major factor contributing to poor cardiovascular health is the lack of sufficient physical activity (PA). The Centers for Disease Control and Prevention reports that 24% of US adults do not engage in leisure-time PA. National averages for women and African Americans are higher, at 29.5% and 26.4%, respectively [3]. A systematic review conducted by Joseph et al found that African American women face unique barriers to PA, including neighborhood safety concerns, financial costs, and hair maintenance, among others [4]. Culturally tailored community-based interventions that combine education, support, and tools to improve cardiovascular health are especially useful when addressing disparities in resource-limited settings [5].

Mobile health technology (mHealth) is a potentially effective and widely accessible platform for community-based interventions seeking to improve health outcomes in areas of lower socioeconomic status. Approximately 72% of African Americans in the United States own a smartphone, and they are more likely to depend on smartphones for internet access than other racial groups. Thus, smartphone apps are a realistic target for interventions among African Americans [6]. As 62% of all smartphone users access health information via their mobile phones, capitalizing on apps to deliver health interventions may facilitate access to communities with lower health care utilization, such as African Americans [7]. This is substantiated by the finding that African American women are willing to participate in mHealth research that uses technology, such as fitness trackers and smartphone apps [8]. To date, there have been no studies that use community-based participatory research (CBPR) methods to engage urban, low-income, African American women as end users in the development of a smartphone app for PA promotion.

In order to understand the potential impact of an app on health behavior, such as PA, it is helpful to rely on an existing theoretical framework. The Health Belief Model (HBM) consists of six relevant constructs related to behavioral change: perceived benefits, perceived barriers, perceived susceptibility, perceived severity, self-efficacy, and cues to action [9]. HBM allows for the systematic exploration of beliefs and attitudes through these well-defined constructs, while other theories [10] do not. Therefore, we propose that HBM will be a strong theoretical basis upon which to analyze the role of an app in facilitating a behavioral change.

Typical CBPR methodology includes working alongside a community advisory board (CAB) to develop a relevant, tailored intervention that is appropriate for the community in question. CBPR, which is focused on the intentional engagement with minority populations, has been a validated approach for eliminating health disparities [11]. Focus groups (FGs) are an effective CBPR methodology for eliciting feedback and gaining a comprehensive understanding of mHealth features preferred by a population. FGs are also at the core of user-centered design processes. Thus, we employed community-based FGs to facilitate user-centered design and develop a smartphone app that promotes PA by addressing the unique needs and barriers of African American women. The primary aim of this pilot study was to employ FGs to qualitatively gather the perspectives of low-income, African American women on a PA–promoting app prior to use in a future, larger mHealth-enabled intervention in the Washington, DC metropolitan area. Applying HBM as a theoretical framework in the thematic analysis of FGs allowed us to effectively explore the utility of the app for improving health behaviors in this population.

## Methods

### Overview of Study Design

A convenience sample of African American women participated in 2 FGs assessing their views on the use of mHealth tools. Immediately following the preintervention FG session, the study app was downloaded onto participants’ mobile phones, and participants were given study-associated Fitbit accounts and a Fitbit Charge 2 device (Fitbit, Inc, San Francisco, CA, USA). Therefore, all participants began both study components—using the app and wearing the Fitbit—simultaneously. After completion of the 20-day study period, participants took part in the postintervention FG interviews to share their experiences with the study app and Fitbit device. The pilot study was conducted for 20 days to accommodate the 18-day push-notification message scheme, plus 1 extra day on each end. Findings of the postintervention FG are described in this manuscript. The study was approved by the National Heart, Lung, and Blood Institute (National Institutes of Health, NIH) institutional review board, and all participants provided written informed consent (NCT 01927783).

### Study Population

African American women aged 19-85 years and residing in low-income areas of Washington DC (wards 5, 7, and 8) and Prince George’s County, MD, were invited to participate. Participants were recruited from a convenience sample of communities in Washington, DC metropolitan area between August 2017 and October 2017. Participants learned about the study through local health education events, flyers at churches, and peer recommendations. Participants were required to own a smartphone device, be proficient in written and spoken English, be physically able to engage in study activities, and be either overweight or obese (body mass index, BMI, ≥25 kg/m^2^) by self-reported height and weight. The study criteria and convenience sampling resulted in the recruitment of 20 possible participants and, ultimately, enrollment of 16 participants.

### Developing the Smartphone App

The app was developed in partnership with Vibrent Health (Fairfax, VA, USA), a health technology company. The app was designed to deliver motivational messages via push notifications, educational content about PA, and a daily self-assessment of stress and participants’ opinions of the message content of that day. The app featured a welcome video of the Principal Investigator (TP-W) explaining the purpose of the study and encouraging participants to increase their PA. The app was tested by the study team and app development group prior to distribution to study participants.

#### Community-Based Participatory Research for Push Notification Message Development

Using the Communication, Awareness, Relationships, and Empowerment Model [12], we engaged our community partners through our previously described CAB, the DC Cardiovascular Health and Obesity Collaborative [13,14]. These community partners provided feedback and input on study design and implementation, including a review of the recruitment and data collection methods.

A questionnaire was emailed to the CAB to identify local and culturally relevant barriers to PA and to solicit suggested motivational push notification messages that promote PA. Follow-up telephonic interviews were then conducted based on a prewritten script to further ascertain and clarify their suggestions (see Multimedia Appendix 1, which illustrates the CAB interview script for suggested messages). Both the questionnaire and interview asked CAB members to provide messages applicable to 1 of the 4 motivational constructs: self-efficacy, self-esteem, goal setting for increasing motivation, and goal setting for those with motivation but limited time. These categories were chosen based on prior work identifying barriers to PA for African American women [15] and the proposed theoretical framework for a community-based, mHealth-enabled PA intervention in development [16]. During the interview, respondents were given examples of messages from each category consecutively to prompt message suggestions relevant to that category. Additionally, CAB members were asked to tailor their suggestions to the population of interest, African American women, in order to generate the greatest impact on PA [17]. These messages were compiled, reviewed, selected, modified, and reorganized into the 4 categories prior to consulting a health communications expert (SE-T) for approval. The final messages were incorporated into a database for use in community-based PA interventions (see Multimedia Appendix 2, which illustrates the process of engaging the CAB for the push notification message development).

#### Motivational Push Notification Message Dissemination

The app was designed to deliver 3 motivational messages daily via push notifications, which included photos of African American women of all body sizes and ages participating in PA. The app also allowed participants to review past messages by storing all received messages on a “wall” within the app. Motivational messages were disseminated via the app according to a programmed order (see Multimedia Appendix 3, which shows the programmed order of message dissemination; arrow represents 1 individual, N=16). Participants were scheduled to receive a specific sequence of motivational messages over an 18-day period split into six 3-day blocks. During each 3-day block, participants were scheduled to receive daily messages from 1 motivational category or to review educational modules. Each participant would, therefore, receive a unique sequence of messages over the testing period to control for sequencing effects, while allowing them to see examples from each motivational category and the educational modules. During the final 3-day block, participants were randomized to receive daily messages from a single motivational construct in combination with the educational modules.

#### Educational Modules

**Figure 1:**
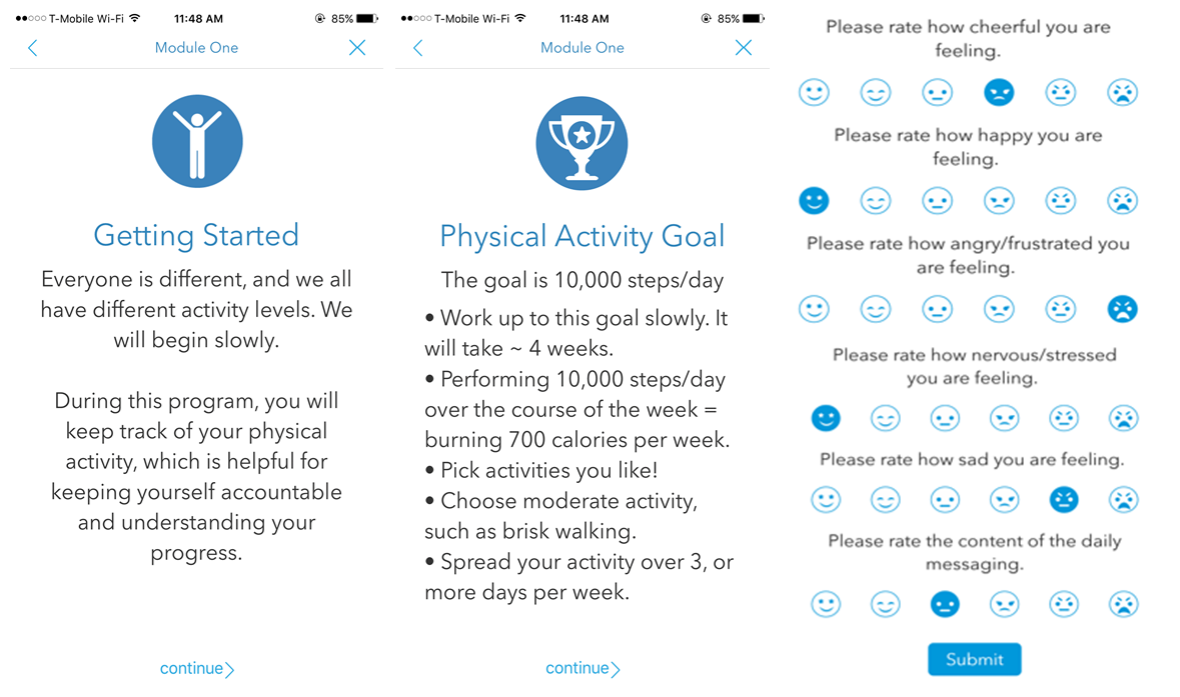
Screenshots of the study app which show Educational Module 1 and the Daily self-assessment.

Information about PA was distributed via 2 educational modules, adapted from the Diabetes Prevention Program (Figure 1) [18]. Module 1 covered goal setting and benefits of PA. Module 2 covered safety, stretching, and how to integrate PA into a busy life. Both modules were interactive, allowing participants to enter their current PA levels and goals for the following week. Upon completion of both modules, participants received further positive reinforcement (a “prize”) within the app, which consisted of 2 simple, heart-healthy recipes.

#### Daily Self-Assessment

Participants were asked to complete a 6-item self-assessment every evening that evaluated their stress and cognitive affect (Figure 1). The first 5 questions were derived from prior ecological momentary assessment tools [19,20]. Participants were asked to rate their levels of cheerfulness, happiness, anger or frustration, nervousness or stress, and sadness on a 6-point scale. For the 6^th^ question, participants were asked to rate the content of the daily motivational push notification messages on a 6-point scale.

### Wearable Physical Activity Tracker

Participants wore Fitbit Charge 2 devices 24 hours per day for the 20-day study period, with the exception of water-based activities. The wrist-worn activity monitor recorded minute-by-minute amount and intensity of PA achieved by each participant as well as sleep duration and quality. As the study app did not sync with the PA tracker, participants were able to view their activity via the commercially available Fitbit app.

### Focus Groups

Immediately following the 20-day study period, all participants met at a local partnering church to discuss their experiences with the PA–promoting app, fitness tracker and associated app, and resulting changes in PA. Then, 2 simultaneous FGs, which included 8 participants each, were conducted to allow all 16 participants greater opportunity to speak. The FGs were conducted using a semistructured interview process. Each FG was led by 2 study team members, a moderator and a facilitator, with additional note takers present to document nonverbal responses. Both moderators followed a Moderator’s Guide, which included preselected questions and probes but allowed for open discussion based on the comments raised (see Multimedia Appendix 4, which illustrates the Moderator’s Guide). During FG, the facilitator’s role was to manage the equipment (ie, name cards and tape recorders), take notes, make observations, and ask follow-up questions when deemed necessary. The FGs were audiorecorded and transcribed verbatim by an independent clinical research organization (Social Solutions International, Inc, Silver Spring, MD, USA).

### Data Analysis

Descriptive statistics were assessed from demographics and Fitbit data using SAS 9.4 (SAS Institute, Cary, NC, USA). A behavioral theory-driven thematic analysis based on the HBM was used to analyze FG data. The transcripts were reviewed, and a preliminary codebook of themes was developed based on theoretical constructs of the HBM. Each theme was accompanied by an operational definition that allowed 3 coders to systematically and independently identify quotes from the FGs that represented each theme. The coding process was iterative, with a total of 6 codebooks developed until consensus was achieved. An intramural NIH qualitative research expert (GW) validated the final coding and corresponding themes.

## Results

### Sample Characteristics

The study sample consisted of 16 African American women with a mean age of 62.1 (SD 6.6) years (Table 1). Mean BMI was 35.5 (range 25.6-54.6) kg/m^2^, with 75% (12/16) of the women classified as obese (BMI ≥30 kg/m^2^). About 63% (10/16) of the women were retired or unemployed. Educational attainment varied and ranged from high school level to graduate or professional degree although most participants (12/16, 75%) had, at least, a college education. Income information was only available for 8 participants, with the majority of participants having a household income ≥US $60,000. Participants had an average step count of 7359 steps per day, with a valid day defined as 10 or more hours of Fitbit wear time. There were 292 valid days for analysis. Step data were averaged over valid days for the 20-day period.

**Table 1:** Sample characteristics (N=16).

Characteristic		Values
Age (years), mean (SD)		62.1 (6.6)
**Sex, n (%)**		
	Female	16 (100)
**Race, n (%)**		
	African American	16 (100)
**Employment status, n (%)**		
	Employed	6 (37)
	Retired or unemployed	10 (63)
**Income (US $), n (%)^a^**		
	<60,000	6 (37)
	≥60,000	5 (63)
**Education, n (%)**		
	Some college or below	4 (25)
	Technical degree	2 (12)
	College degree	7 (44)
	Graduate or professional degree	3 (19)
**Marital status, n (%)**		
	Single, divorced, or widowed	12 (75)
	Married	4 (25)
**Location of residence, n (%)**		
	Maryland	7 (44)
	Washington, DC	9 (56)
Body mass index (kg/m^2^), mean (SD)		35.5 (8.29)
**Weight parameters**		
	Overweight^b^, n (%)	4 (25)
	Obese^c^, n (%)	12 (75)
**Physical activity parameters, mean (SD)**		
	Steps per day^d^	7359 (2201)
	Sedentary minutes per day	1174 (54)
	Light intensity minutes per day	236 (50)
	Moderate intensity minutes per day	12 (8)
	Vigorous intensity minutes per day	18 (10)

^a^Income information was only available for 8 participants.

^b^Body mass index≥25 kg/m^2^.

^c^Body mass index≥30 kg/m^2^.

^d^Valid day defined as ≥10 hours of wear time (step data averaged over valid days during the 20-day study period).

### Focus Group Themes and Subthemes

The HBM includes 6 constructs, of which the following 5 were germane to the data: perceived benefits, perceived barriers, perceived susceptibility, cues to action, and self-efficacy. Perceived severity was not identified in the transcripts. Additional, emergent themes were identified, including technical difficulties, generational differences, and relationship with the community (Textbox 1). Themes, subthemes, and illustrative quotes can be found in Multimedia Appendix 5. As the app was designed to provide multidimensional behavioral support, instances of overlap between themes arose during the analysis, for example, between perceived benefits and cues to action as well as perceived barriers and technical difficulties. However, many quotes succinctly and fully illustrated a single theme, such as the following perceived benefit:

I didn’t lose weight, but it showed in my blood tests; the results of my blood tests. So, I did show some improvement with the increasing of the exercise.

This participant is highlighting the benefit she perceived from using the app to increase her PA (improved laboratory tests), in the absence of achieving her personal goal (weight loss).

### Participant Suggestions for the App Improvement

During the FGs, participants shared their opinions of the app and provided suggestions for further improvement (Table 2). Participants strongly expressed a desire for increased educational content, more tailored location-specific recommendations for outdoor PA, and in-app connection to existing community resources that may assist in overcoming barriers to PA. They also suggested that the app show PA data from the Fitbit device (rather than on the separate, Fitbit app), include personalized step goals, and have a redesigned daily self-assessment tool.

**Themes and subthemes.**

**Health Belief Model**

- Perceived benefits
  - Impact on nonphysical activity health behaviors (mood, sleep, healthy eating, etc)
  - Goal setting
  - Education or new information
  - Safety of global positioning systems tracking
- Perceived Barriers
  - Difficulty of use (ie, lack of automation)
  - Ambiguity over goals of daily self-assessment
  - Accuracy of physical activity tracking or ambiguity over physical activity goals
  - Technology literacy
  - Community or historical distrust of research
  - Safety as a barrier to physical activity
  - Insufficient data plan or memory
- Perceived Susceptibility
- Cues to action
  - Push notification messaging
  - Expanding the definition of “exercise”
- Self-efficacy

**Emergent Themes**

- Technical difficulties
  - Check-ins or IT support
- Generational differences
- Relationship with community
  - Connection to fellow participants
  - Connection to the research team
- Social support
- Preferred features

**Table 2:** Participants’ suggestions for the app improvement.

Suggestion		Illustrative quotes
**Increased automation**		
	Water intake	“So if I drank the water and it automatically like it does my sleeping. It counts the sleep. It needs to count the water.”
	Food intake	“The food intake. I put in one thing and then I said, ‘Oh wow, this is just, I don’t know how much this is.’[…]But I looked at that and I said, ‘This is a little too much [work].’”
**Increased relatability**		
	Redesign the daily self-assessment	“We not no children, get rid of them [smiley faces].” “I’d definitely get rid of them smiley faces. I didn’t like the smiley faces. I thought [they]made me feel like I’m in the elementary with my grand[daughter].” “I couldn’t quite relate to the daily self-assessment, the rating scale, the happy faces. I think it was more like giving each segment stars, I could have understood that better. I couldn’t relate to [the smiley faces].” [Speaking about revising the scale in the self-assessment] “Like on a scale from 1 to 10, on a scale from 1 to 10, I’m like a 7 or…”
	Including photos of heavier women	[Speaking about the photos that accompanied the messages] “Have a person that’s heavier than these people. They look like they’re already fit.” “I liked, well, when I read I like to see people that look like me.” “It’s really important that I see somebody with some gray hair…” “Weight.” “Heavy!”
**Ease of use**		
	Saving or printing the recipes	“And then you’re on your phone and you don’t have a printer see you’re not putting it on your iPad or anything. If you had that you’d print it out. I couldn’t print it out and I wasn’t going to handwrite it.”
**Increased educational material**		“Even add a few more modules.”
	Diet and nutrition	“About I guess they could talk about, because I know a lot of people don’t like calorie counting and different things but the health coach that I’m working with, or the program that I’m working with, we have measurements and they show you what your size is you know, protein your palm of your hand, the grain your first, or different things like that and they do a lot of visuals and so on like that so that we don’t have to worry about counting and how to prepare healthy nutritious meals you know with a protein, a carb I mean your grain, your vegetable. Things like that.”
	Stretching, including an instructional video	“If there’s a video like there the pictures may show you someone exercising, how to exercise. Maybe if there is a video if the video shows you how to do that exercise or proper technique for that exercise or the benefit of that particular exercise. If the if video was to be included I think that would be helpful.” “I agree because I remember…they were saying roll your flatten your back to the floor and I kept trying to visualize what they were saying. So, maybe a video would have helped.” [Discussing stretching techniques shared in the educational module.]
	Sleep hygiene	“Maybe in the future since there are some of us that have sleeping issues. Maybe I didn’t see anything at any of the questions, like suggestions about either going to bed early, turning the T.V. off, you know those kinds of suggestions to help us.”
**Connection to community resources**		“I’m thinking maybe you know safe places to exercise in the community, places where we can get food, you know just whatever. Any kind of resources that I think would pair well with exercising and eating well and taking care of yourself.” “[I] think you could maybe add somewhere, I don’t know where but maybe somewhere more information about resources.” “A cooking class.” [Speaking about community resources she would like to see included in the app]
	Group exercise or dance classes at existing facilities	“Yeah, group exercise and stuff like that. And you’d get more people motivated and doing this cause so far that’s what we’re doing at the [Recreation Center]. We’re spreading the word and we’re getting a lot of people coming in now…The classes are free and they giving them four days a week. You could do stuff up there, and the most time they got line dance, hand dance, jazz you know all these classes.”
	Community walking groups and ability to create them in the app	[Speaking about the benefit of having a group or competition component within the app] “Yeah, I know and that’s what made me think within the study maybe they could do something like that. Even if it’s to link up with some of those that is already in existence.” “You don’t like to walk alone because it’s not safe nowadays. But, if there was some kind of information that would say like, ‘Look, we would like a group to walk, a start off group to walk at five o’clock in the morning in a certain area.”
	Safe places to walk or exercise outside	“Yes, and I think we just did a thing on, what they call it? Geographical information systems where they show you know they map out areas where you can go to get physical activity and different things like that. Because you know health information is being done through a lot of technology and that would be one because sometimes people in the community need to know where can I go, you know how can I get there? For children through adults. Different things like that.”

## Discussion

### Principal Findings

This study demonstrates the effectiveness of community engagement techniques, such as CBPR and FGs, in developing and refining culturally tailored smartphone apps for use in a community-based, mHealth-enabled PA intervention. Introducing a user-centered mHealth intervention in a low-income, African American population at risk for cardiovascular disease increases the likelihood of adoption and, therefore, may be an effective method for addressing health disparities.

Despite the purported potential of technology to alleviate health disparities, little research has been done to evaluate the feasibility and effectiveness of mHealth in minority populations. Consistent with recent studies, we show that both wearable technology and smartphone apps are well received among urban, community-dwelling African American women [8,13]. Location-based tracking was not perceived as a significant barrier for the adoption of technology-based interventions in this population. Rather, it was seen as a benefit due to the possibility of locating an individual in the event of safety concern (ie, abduction). In summary, mHealth may be a useful tool for promoting PA among minority and low-income populations, and future work to evaluate its efficacy is warranted.

#### Community-Based Participatory Research for User-Centered Design

Involving community members at the earliest stage of the study design upholds the essence of true CBPR methodology. We were able to incorporate the community perspective directly into the intervention through the process of developing tailored motivational messages with the CAB. The majority of CAB members share residence and demographic characteristics with the study participants. This allowed us to overcome a strong barrier to effective health disparities research, namely, the predominating influence of an “outsider” perspective. Previous research demonstrates the effectiveness of culturally relevant persuasive messages for PA promotion among African American women [21,22]. Effectively engaging the community through CAB allowed us to generate culturally tailored messages, which also highlighted unique barriers to PA for this population, such as safety and hair maintenance. The success of this collaboration allowed us to better address the needs of the study population, while simultaneously empowering the target population as a mutual partner. Therefore, we see the formation of CAB to work alongside health disparity researchers as a necessity.

#### Focus Group Interviewing for User-Centered Design

Using FGs to inform the development of our PA app was an effective method to facilitate end user tailoring and, potentially, satisfaction. Although prior studies have used FGs for mHealth interventions, most explored end user satisfaction and feasibility only after the final app development and immediately before commencing a randomized control trial. Very few studies have used FGs as a means of collaborating with the target population to obtain feedback for the enhancement of a PA app at its inception [23-28].

#### Focus Group Interviewing and the Health Belief Model

Grounding our qualitative data analysis within HBM allowed us to identify how the app acted as both a promoter and barrier to behavioral changes. Specifically, HBM elements showcase the app’s potential to facilitate a behavioral change by means of educating users on the benefits of PA, promoting self-efficacy, and providing cues to action. Although discussion on perceived severity did not arise during the interviews, that particular construct has been shown to be less influential in facilitating a sustained health behavioral change [29,30]. Conversely, the concept of perceived susceptibility was raised during the discussion and has been shown to play a significant role in developing health behaviors [31]. A concern that often arises in the dialogue surrounding minority health and research participation is the historical and pervasive mistrust of the scientific community [32]. Indeed, participants discussed the potential of community distrust to act as a barrier to app adoption. However, they also highlighted and reinforced CBPR as a means of reducing hesitancy and enhancing trust. In summary, HBM constructs were especially useful in informing the development of an mHealth-based intervention, which aims to increase PA as a preventive health behavior in limited-resource minority communities.

#### Tailoring the App to User Needs and Preferences

While the primary focus of the intervention, PA, was well received by participants, the qualitative data demonstrate a need for a significant secondary impact on nonPA health behaviors. Participants expressed a strong desire for additional information regarding other beneficial lifestyle modifications. For example, they spoke frequently about increased awareness of their sleep patterns and need for improved sleep hygiene. Discussion on diet and nutrition was also extensive, including a desire for increased information on portion size, healthy choices, and water intake. Participants also discussed their novel awareness of the connection between mood and activity. They expressed a realization that exercise can improve mood and discussed the benefits of engaging in relaxation and mindfulness techniques. Increased awareness of benefits of PA, including mood, may generate an additional, novel reinforcing mechanism that increases the probability of future PA.

Similarly, social support was a recurrent theme, and the participants’ comments suggest a relationship wherein social support is a vehicle for increased self-efficacy. Improved self-efficacy has been shown to substantially increase the probability of successful maintenance of health interventions, including smoking cessation [33], PA [34,35], and weight loss [36,37]. Participants’ social networks, including accountability of partners, family, and key community members, were repeatedly juxtaposed with expressions of self-efficacy. Therefore, incorporating social networks in future interventions may improve self-efficacy and encourage sustained behavioral changes. Participants also referenced several opportunities for increasing social support from within the app, including the formation of competition and walking groups. They expressed the desire to be able to communicate and share resources with fellow community members, which was not an available feature within the app. Additionally, they described the relatability of the app content (ie, photos of heavier and older women engaging in PA) as a form of social support. Finally, they viewed the technology itself (Fitbit) to be a common ground around which they could form groups and connections within the broader community, for example, at local recreation facilities. Capitalizing on the broad impact of social support on motivation, self-efficacy, and adherence may further promote sustained behavioral changes among urban African American women.

Our findings affirm that using FGs to identify values, goals, experiences, and definitions of PA may result in more informed and, therefore, more effective strategies for PA promotion [28]. Having engaged the population of interest early in the process of app development allows us to incorporate key suggestions into future iterations of the app and, therefore, build the most accessible, efficacious platform for increasing PA within this community. As a result of participants’ feedback, we will modify the app to increase user satisfaction by incorporating information about local parks and fitness classes. We will also increase the user relatability by redesigning the daily self-assessment and adding additional photographs that reflect the cohort’s demographics and physical appearance. Finally, we will incorporate additional educational modules that reflect participants’ desire for information on nonPA health behaviors.

### Limitations

As this was a pilot study, the duration of the intervention was short, and the sample size was small. The study population was a convenience sample of middle-aged, African American women who were recruited from communities within Washington, DC metropolitan area. Although our findings may not be generalizable to other populations, they may be generalizable to other African American female residents of urban environments. The use of the Fitbit PA app may have influenced participants’ perceptions of the study app, but this was not assessed. Finally, due to technical difficulties, some participants were not able to access the entire app content, and we were unable to objectively evaluate the influence of the push notification message dissemination protocol on PA.

### Comparison With Prior Work

Previous work has explored smartphone usage and willingness to participate in mHealth weight management research via quantitative data collection [6]. However, this study is the first to employ FGs for qualitative data collection of such information. Furthermore, this study is the first to employ FGs to guide the development of a culturally tailored app for a community-based, mHealth-enabled PA intervention among African American women. The findings from this FG reaffirmed previously cited barriers for African American women for engagement in PA [4].

### Future Directions

Although culturally tailored push notification messages were the focus of this study, we plan to expand this approach to include geographically and personally tailored push notification messages as well. For example, the use of geographic information systems can provide suggested locations for safe sites for outdoor PA and increased awareness of existing community PA resources, such as recreation centers. Incorporating personally tailored step goals and push notification messages based on the real-time activity may further promote sustained PA as it has been shown that personalized, adaptive goal setting improves adherence to PA interventions [38,39]. It is thought that fixed, nonpersonalized PA goals can be discouraging to participants as goals may be either unrealistically high or, conversely, not adequately challenging [40-42]. Personalized PA goals can be accomplished through machine-learning techniques, as recently demonstrated by Zhou et al [42]. Incorporating these techniques in the future app development may strengthen user satisfaction and effects of the intervention.

### Conclusions

This pilot study demonstrates that the development of mHealth-enabled interventions based on the qualitative CBPR methodology and community member engagement may improve future PA and cardiovascular health interventions. The resulting enhancements to the app may be useful in ameliorating health disparities and improving health outcomes of underserved, minority communities by increasing the likelihood of acceptability and utilization of mHealth by target users.

## Appendix

Multimedia Appendix 1 Community Advisory Board interview script for suggested push notification messages.

Multimedia Appendix 2 Diagram of the motivational push notification message development process.

Multimedia Appendix 3 Diagram of the pilot study push notification messaging strategy with educational modules.

Multimedia Appendix 4 The Moderator’s Guide.

Multimedia Appendix 5 Focus group themes, subthemes, and quotes.

## Abbreviations

BMI: body mass index
CAB: community advisory board
CBPR: community-based participatory research
FG: focus group
HBM: Health Belief Model
mHealth: mobile health
NIH: National Institutes of Health
PA: physical activity
