# Supplementary material for: Community Engagement in the Development of an mHealth-Enabled Physical Activity and Cardiovascular Health Intervention (Step It Up): Pilot Focus Group Study
Source: JMIR Form Res. 2019 Jan 4;3(1):e10944. doi: 10.2196/10944 (PMC6682281; doi:10.2196/10944)
Supplement: Multimedia Appendix 1 [file formative_v3i1e10944_app1.pdf]

<table>
<tr><td colspan="2">Community Advisory Board Interview Script for Suggested Messages</td></tr>
<tr><td colspan="2">Introduction</td></tr>
<tr><td colspan="2">Hi (CAB member)! As you might be aware, the Powell-Wiley group is developing a tailored mobile intervention to increase physical activity among African American women in the community. We are currently gearing up to create a bank/pool of messages that would encourage participants to do more physical activity at home, work, church, or anywhere else that they may be. We want these messages to reflect what our community wants to see, which is why we are asking for your feedback.
We identified 4 broad categories of messages that we will send to women in our study. I will read a description and example of each type of message. And what I would like you to do is come up with as many short messages that you think fall under each category.</td></tr>
<tr><td colspan="2">Category 1: Goal Setting Messages</td></tr>
<tr><td>Purpose</td><td>Probes for Suggestions</td></tr>
<tr><td>People have extremely busy lives. This category of messages is to help those people set general goals for themselves so that they can be physically active and provides them with specific steps that they can take to achieve these goals!</td><td>Can you think of example messages with reasonable goals for physical activity/getting in shape for members of the community?
(Probe for three to four goals.)
Example: “Aim to walk 10000 steps today.”

Can you think of example messages with actionable plans to help members of the community get physically active?
(Probe for three to four action plans related to the goals identified in the previous question.)
Example: “Reduce your time sitting. Suggest a walking or standing meeting at work.”

What would you tell a friend who is struggling to set a physical activity goal and reach it?
Example: “You don’t have to walk 10,000 steps all at once, aim for 1000 steps in your first week of committing to being active.”</td></tr>
<tr><td colspan="2">Category 2: Self-Efficacy Centered Messages</td></tr>
<tr><td>Purpose</td><td>Probes for Suggestions</td></tr>
<tr><td>With these messages, we want our community members to know they can do it! They can be physically active despite any barriers.</td><td>Can you think of reasons as to why an African American woman in the community may not be physically active? The most common reasons are lack of time or money. Can you think of anything else?

What are some of the messages that you think can boost women’s confidence in being active?
(Probe for messages that counter the barriers identified in the previous question.)
An example, here, can be: “Step by step, you can reach your physical activity goals for today.”

“Even if you don’t have access to a gym, you can</td></tr>
</table>

	exercise at home. Running in place or jumping jacks increase your heart rate and are considered high-intensity exercises."
Category 3: Motivational Messages	
Purpose	Probes for Suggestions
Although most people know it is important to be physically active, some might not be motivated to do so.	What are some of the reasons that you would use to encourage them to be physically active if they are unmotivated to do so?<br>(Probe for three or four messages.)<br>Example: *"Being physically active means more time spent playing with your children."*<br>*"When you are physically active, you are in a good mood."*
Category 4: Self-Esteem Boosting Messages	
Purpose	Probes for Suggestions
Okay (CAB member!), this is the last category of messages. With these messages, we want to reward our community members for sticking with their goals. Also, we want to encourage those who did not meet their goals or fell off to start again.	For example, a participant might receive this message: *"Good job maintaining your weekly step total! - keep it up :-)."*<br>*"Congratulations! You have continued to increase your steps throughout the week!"*<br>*"You did great this month. You deserve a massage or a pedicure!"*<br><br>Can you think of any words of encouragement/rewards for our community members who stay on track?<br><br>Can you think of any messages for the community members who did not stay on track?<br>An example can be: *"A little set back in this week's goals is not the end of the world. Start again tomorrow."*
Closing	
Thank you so much for taking the time to complete this survey. We really appreciate your contribution!	

This is a Multimedia Appendix to a full manuscript published in the J Med Internet Res. For full copyright and citation information see http://dx.doi.org/10.2196/jmir.10944
