# Supplementary material for: Community Engagement in the Development of an mHealth-Enabled Physical Activity and Cardiovascular Health Intervention (Step It Up): Pilot Focus Group Study
Source: JMIR Form Res. 2019 Jan 4;3(1):e10944. doi: 10.2196/10944 (PMC6682281; doi:10.2196/10944)
Supplement: Multimedia Appendix 2 [file formative_v3i1e10944_app2.pptx]

## Slide 1
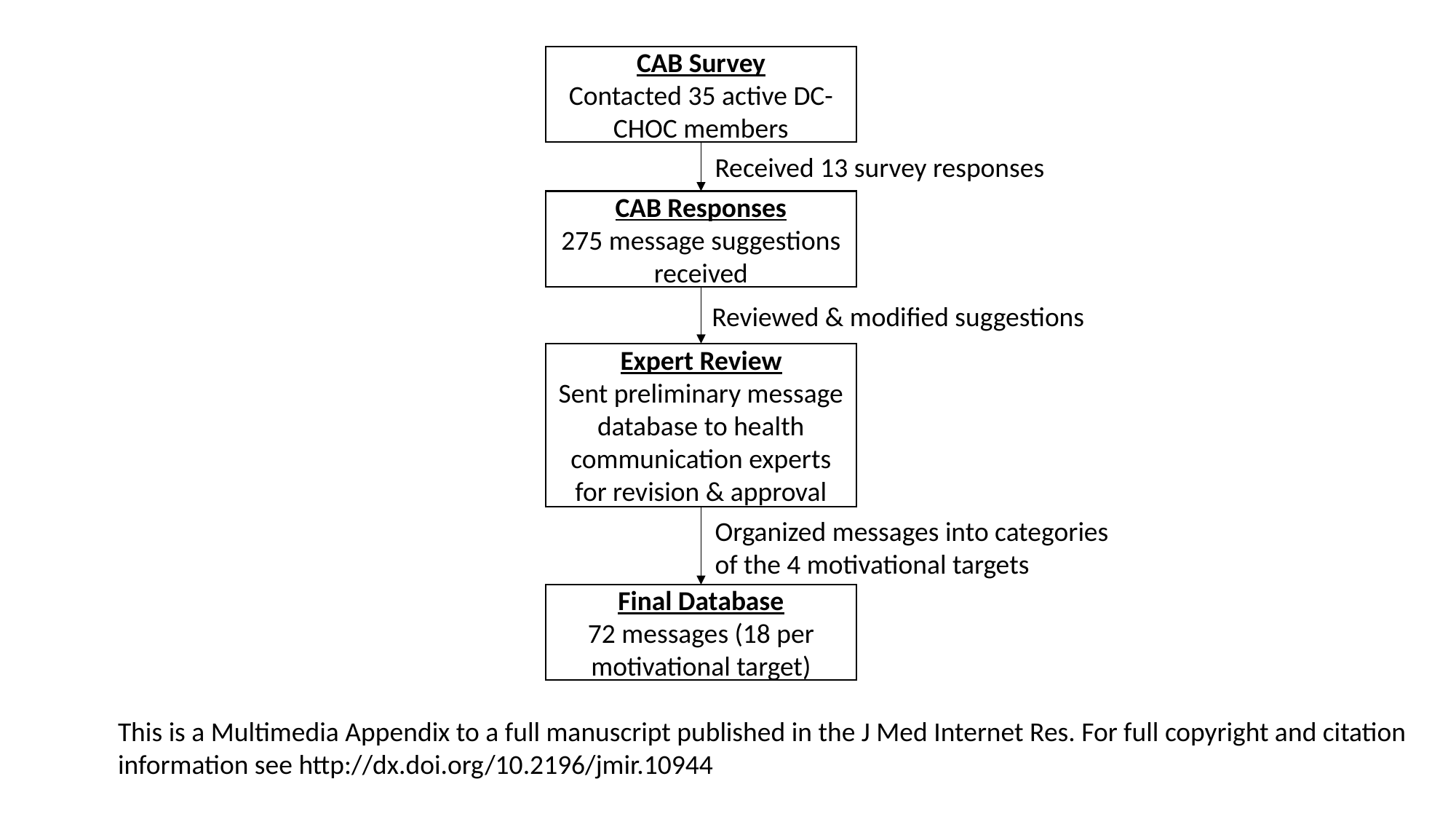

CAB Survey
Contacted 35 active DC-CHOC members
Received 13 survey responses
CAB Responses
275 message suggestions received
Reviewed & modified suggestions
Expert Review
Sent preliminary message database to health communication experts for revision & approval
Organized messages into categories of the 4 motivational targets
Final Database
72 messages (18 per motivational target)
This is a Multimedia Appendix to a full manuscript published in the J Med Internet Res. For full copyright and citation information see http://dx.doi.org/10.2196/jmir.10944
