# Supplementary material for: Community Engagement in the Development of an mHealth-Enabled Physical Activity and Cardiovascular Health Intervention (Step It Up): Pilot Focus Group Study
Source: JMIR Form Res. 2019 Jan 4;3(1):e10944. doi: 10.2196/10944 (PMC6682281; doi:10.2196/10944)
Supplement: Multimedia Appendix 3 [file formative_v3i1e10944_app3.pptx]

## Slide 1
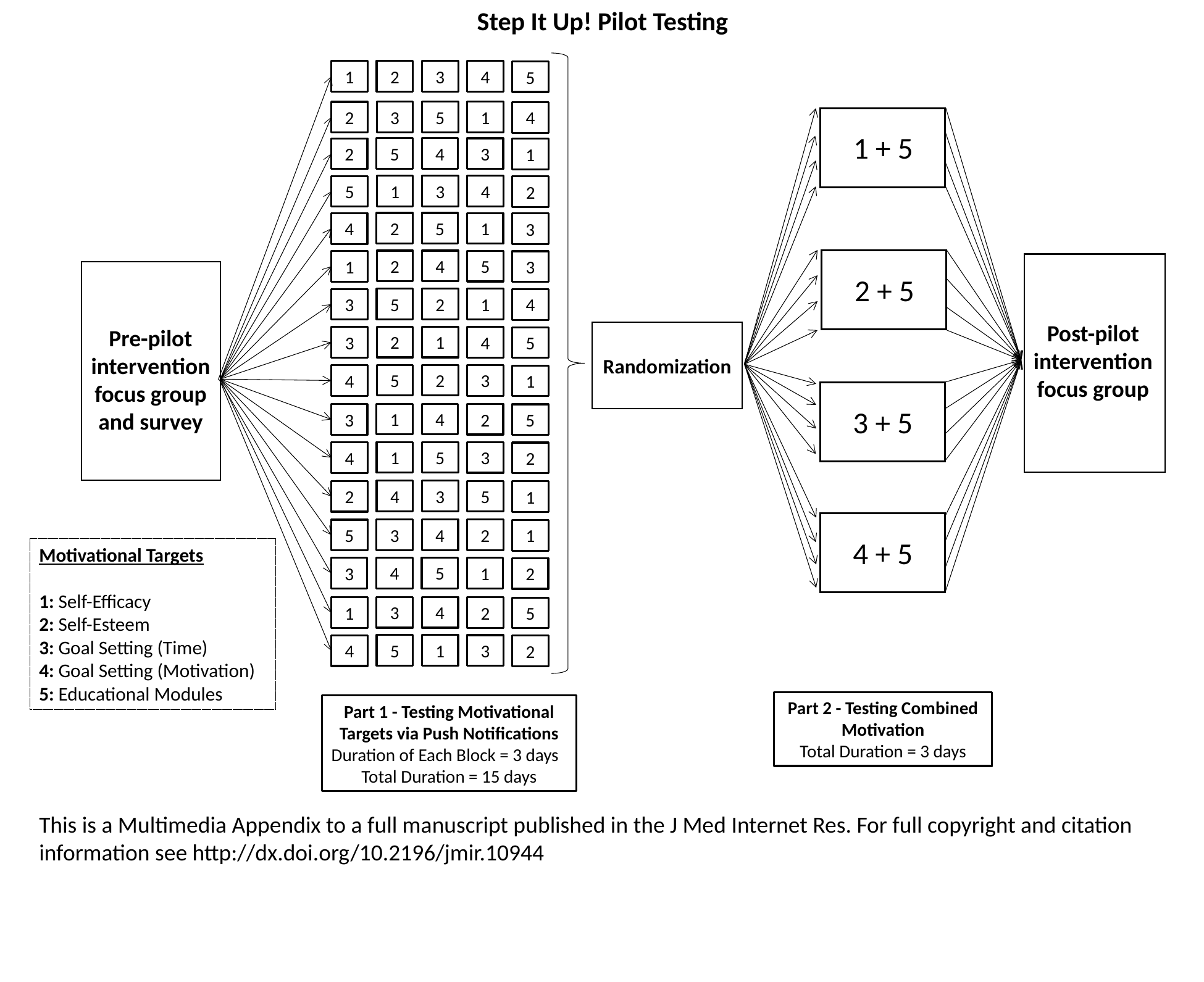

Step It Up! Pilot Testing
2
3
4
1
5
3
5
1
2
4
1 + 5
5
4
3
2
1
1
3
4
5
2
Post-pilot intervention focus group
2
5
1
4
3
2
4
5
1
3
2 + 5
5
2
1
3
4
Pre-pilot
intervention focus group and survey
Randomization
2
1
4
3
5
5
2
3
4
1
3 + 5
1
4
2
3
5
1
5
3
4
2
4
3
5
2
1
3
4
2
5
1
4 + 5
Motivational Targets
1: Self-Efficacy
2: Self-Esteem
3: Goal Setting (Time)
4: Goal Setting (Motivation)
5: Educational Modules
4
5
1
3
2
3
4
2
1
5
5
1
3
4
2
Part 2 - Testing Combined Motivation
Total Duration = 3 days
Part 1 - Testing Motivational Targets via Push Notifications
Duration of Each Block = 3 days
Total Duration = 15 days
This is a Multimedia Appendix to a full manuscript published in the J Med Internet Res. For full copyright and citation information see http://dx.doi.org/10.2196/jmir.10944
