# Supplementary material for: Community Engagement in the Development of an mHealth-Enabled Physical Activity and Cardiovascular Health Intervention (Step It Up): Pilot Focus Group Study
Source: JMIR Form Res. 2019 Jan 4;3(1):e10944. doi: 10.2196/10944 (PMC6682281; doi:10.2196/10944)
Supplement: Multimedia Appendix 4 [file formative_v3i1e10944_app4.pdf]

**Supplemental Digital Content 3. Moderator's Guide**
Icebreaker – Participants were given screen shots of the application and a list of all the motivational messages.
Mark your favorite aspect of the app, redesign an element of the app, or add anything that's missing.
Place a star next to your top 3 messages on each page.
Application and Fitbit Follow-up
1. What were the hardest instructions to follow and why? What were the easiest instructions to follow and why?
2. In what ways did the app encourage you to increase your physical activity? In what ways did it discourage you, or feel unhelpful?
3. What did you think of the timing of the messages and when they were delivered?<br>Probe: Too many, too few, inconvenient times, etc.
4. In general, what did you think of the content of the messages?
5. How did you feel about the photos that accompanied the messages?
6. What were the overarching themes you identified within the messages? Which were helpful, not helpful? How did they help, not help?<br>Probe: Encouraging, goals, tips, etc.
7. Was there a specific message that stood out to you?<br>Probe: Describe how that message was good/bad, or how it made you feel.
8. What did you think about the message-board, or wall?<br>Probe: Did you find it helpful? Repetitive?
9. What did you think about the educational modules?<br>Probe: Content? Length? Format? Suggestions for improvement?
10. What did you think about the prize recipe?<br>Probe: How did it make you feel? Did you make the item?
11. What did you think about the daily assessment?<br>Probe: Too many questions? Inconvenient? Suggestions for improvement?
12. Please tell us about which features you liked on the Fitbit app.<br>Probe: Ease of use? Prizes? Competition with other users?
13. What problems or issues did you have using the device or app in the last few weeks?
14. What difficulties or issues did you have with accessing the app content from your devices?
15. What difficulties did you have with the activity tracker/wristband (Fitbit)?<br>Probe: Issues with syncing? Charging?
16. Did you have any skin irritation, rash, pain, or physical issues while using the Fitbit? How bad were those issues?
Wrap Up
17. What are your overall thoughts and feedback regarding the app?<br>Probe: Recommendations/suggestions for feasibility?
18. Who do you think would be most likely to use this app in your community?<br>Probe: People who want to lose weight, increase their activity, or improve overall health
19. How do you think a member of your community would respond to receiving this app and activity tracker?<br>Probe: Concerns about data plan, tracking, etc.
20. What do you see as potential barriers for people using this app in your community?<br>Probe: Data plan, comfort level with technology, time, childcare, etc.
21. Would it be valuable, or unnecessary to offer these activity trackers and apps in your community? Explain.
22. Do you have any suggestions for other things you would like to see or use if this app was offered to you and others in your community?
22. Is there anything we have not discussed that you would like to share?
