# Supplementary material for: Community Engagement in the Development of an mHealth-Enabled Physical Activity and Cardiovascular Health Intervention (Step It Up): Pilot Focus Group Study
Source: JMIR Form Res. 2019 Jan 4;3(1):e10944. doi: 10.2196/10944 (PMC6682281; doi:10.2196/10944)
Supplement: Multimedia Appendix 5 [file formative_v3i1e10944_app5.pdf]

Multimedia Appendix 5. Focus group themes, subthemes, and quotes.

Themes and Subthemes			Illustrative Quotes
**Health Belief Model**			
	**Perceived benefits**		"I didn't lose weight but it showed in my blood tests; the results of my blood tests. So, I did show some improvement with the increasing of the exercise."
		Impact on nonphysical activity health behaviors (mood, sleep, nutrition, etc)	"Well me, I like to monitor, okay. So, I like the part the sleep cause that's one of my things that I'm working on with my health coach too. I'm still struggling with that.[…] But with this I able to see exactly what type of sleeping I'm having."<br><br>"The one, 'Did you know exercise can elevate your mood and decrease your risk for depression?' I just think that's a good one because it does elevate your mood, for me anyway. Rather than just you know sitting at my desk if I move and go outside, I'm an outdoors person anyway, it does elevate my mood. Even just ten minutes."<br><br>(Speaking about daily self-assessment) "Checking it every day and helping to like see where I'm at. Whether I was happy, sad, or whatever because some days I had pain issues and you know, I wasn't happy because I was dealing with pain. And you know just to help me keep track."
		Goal setting	"The thing is they'll tell you to go 15 minutes one way then come back that's 30 minutes. If you walk somewhere 15 minutes, turn around, and walk back 15 minutes. Thirty minutes a day and then I think you do that for about a week, maybe two weeks when you first start out. Then, you progress maybe add 15 more minutes to it or maybe you might do it a month. So, that's what I mean little by little. You don't want to go out there and try to hurt yourself."<br><br>"I'm out there walking and I say, 'Oh, I can walk a little bit more. I'll make my 10,000 steps.' It tells you your steps and

			your heart rate and everything and when you’re walking. So, I like that that it’ll motivate me because I set my goal and most times I met my goal or went over the goal. But I went in there and set the goal that I want to do today or what I thought was manageable for me. […] ‘I’m going to get my 6,000 steps that I said imma do today.’ And then I go on the treadmill, do mine, and I come back and I’m like, ‘I got it!’ and then I just I’m happy, but I could do more. But I set my goal.”
		Education or new information	“Like the proper shoes to wear. I was like shocked when I see that in there. I was like, ‘Okay what’s wrong with my tennies?’ That’s why I said, ‘Let me switch up shoes.’ But, yeah that was really they tell you what type you should wear. You know so you won’t really hurt yourself. Even when they did the one for stretching. The different stretches… the one I had because they had one where you stand up and stretch against the wall. I was like, ‘Oh! Okay!’ Yeah.”<br><br>“I realized I don’t sleep the entire night and I got less than four hours sometimes because you know I’m turning the television off and the house too quiet. So, I turn it back on and I start watching it… but I try I sleep with blinders on. So, that I can still hear the television. But I go into that deep sleep early in the morning so I was you know I like that because I could see my pattern for the past three weeks and stuff. I like really like that. And the water. It forced me to drink more water but start earlier and cut it off around six. Cause you see that was a lot of getting up during the night. Drinking water too late so that all helped.”
		Safety of global positioning systems tracking	“I sort of agree with you but anybody that have a smart phone or any kind of you know (P: Yeah, you’re being tracked all over.) somebody is tracking you. And if I’m abducted I want somebody to… I’m just saying I think I’ve gotten used to it and I don’t really care unless you have a stalker or something

			but everybody with a cell phone they can find you… So, I don't see that as a bigger barrier for people."
	**Perceived Barriers**		"Some of our seniors have flip phones and I was one. I love my flip phone. So, you can't do this with a flip phone. So, it just depends upon what type of communication tools they have."
		Difficulty of use (ie, lack of automation)	"The food intake. I put in one thing and then I said, 'Oh wow, this is just, I don't know how much this is.' I know, I know how to measure like ¼ cup of rice, brown rice or something like that because I've gone to a nutritionist in the past, but I looked at that and I said, 'This is a little too much.' I put I think I put in one chipotle black bean burger and that was it."<br><br>"I wish, I wish it was automatic. So, when you drank the water it counted it."<br><br>"I used that a couple of days and then I kind of got off track and I said, 'I can't put everything down.' I felt kind of guilty too about some of the stuff I was eating."
		Ambiguity over goals of daily self-assessment	"I thought I couldn't distinguish between cheerful and happy. I kept saying, 'Is this the same question? Are they asking me the same question twice?' So, yeah I couldn't really distinguish the two and the smiley faces...I kept thinking, 'The questions are really similar. I don't know how…Is that first question different from the second question?' They just seemed like the same question to me for some reason…<br><br>"'Was I supposed to be stressed?' 'Was I supposed to be nervous some point in time?' And I'm like oh it ain't apply to me. So, I'm like, 'Well, what to put? You know, how should I rate it?' When it's like, 'Well, that didn't even apply to me today.' You know, I didn't have a bad day. I didn't have a stressful day, I wasn't nervous about anything."

			"How to get the nervous part in. You know I was starting to feel like is this supposed to happen throughout the day? Or some point in time throughout the day? Was that supposed to happen to me?"
		Accuracy of physical activity tracking or ambiguity over physical activity goals	"The only thing I didn't like was that I'm in the water and I'm doing intensive activity, but it's not really recording what I'm doing. But I could walk and I'm doing less intensive and I get more steps walking then I do vigorous activity. So, it appears as though I'm not reaching a goal and actually I'm exercising more intensely in the water than I did on land."<br><br>"I want to see results so to me the intensity is greater than the steps. So, it didn't matter, it doesn't record my intensity even though I could run out there and walk…I jumped in the house and I did the rest of the steps."<br><br>"See you do 10,000 steps a day, but then it says, if you read more it says if you do so many hours or 2.5 hours of pro physical activity a week. Right? So, they're saying that 10,000 steps a day which about 150 minutes or 2.5 hours of physical activity. So, if you do 2.5 activity like walking a week, so you don't have to do the 10,000 steps a day?"
		Technology literacy	(Discussing the manual logging of food.) "At least if they gave us clear instructions how we go in and do it. You know like 'Step one. Push the man's face. Okay, step two. You know, do this. You didn't write the list of foods. Type this.' You know what I'm saying. I had it, but you know I was just picking and putting and push and wishing. So, that's how I did it."<br><br>"Okay, so like for you, you said you weren't used to the technology and all of that, so education has to be a part in that how to use stuff. Having somebody to help them along for the

			beginning until they get used to it. So, depending on the age, the simplicity of it, where it’s not too cumbersome, you know would help. So, it depends on the age group.”<br><br>“I wasn’t charging it correctly it has to be upside down or something but anyway I saw that the Bluetooth was on so I turned it off. I think there should be some instructions… because I’m not all that tech savvy. But the two work together you know wireless and so, I went to read my instructions and I said, ‘Oh, the Bluetooth got to be on’ and I still couldn’t…The Fitbit was on the charger for maybe two hours. I went out came back, so I asked my daughter to look at it and she looked and read it and she said, ‘Ma, I think it’s upside…you got to turn it around.’ As soon as she did that the battery came up.”
		Community or historical distrust of research	“And I think one of them it should maybe come up with a cyber technology maybe trust because you know I’ve said a couple of times jokingly, ‘Hmm…I think NIH is tracking my steps.’ And everyone says, ‘Whose tracking you? What’s going on? You’re being tracked?’ because I did a study earlier this year and they were going to people giving them a complete physical exam...And they did not want to go.. So, I kept saying, ‘You’re getting a little money, you’re getting a free examination. Way more exams than you were going to get from your doctor’s office.’ But they, ‘Uh uh, I don’t trust that, that’s the government. I’m not sure if they taking my blood, what do they want to know...’ But even having a FitBit that is not as invasive as those exams are it still could be some trust issues, ‘What are they tracking?’ I was in Atlanta and you called me to say ‘You’re Bluetooth isn’t on,’ that’s a red-flag like how could you know what’s going on with my Bluetooth and I’m 700 miles away from here. So, trust could be the issue with certain people.”

			“I think it depends on how you present it and who you present it through because when I was here the first time, I don’t remember the lady’s name, but she seemed like the, I don’t want to call her like that, leader for lack of a better word. She was the guide and was probably instrumental in bringing a lot of other women to the study... It depends if you go to that person in the community, it could be the person in the church, it could be the person whose leading the groups to exercise. If you go to those individuals and have them present it to the community it might be better well taken.”<br><br>“I don’t think it’s just how it’s presented but also who presents it, I think that that’s really important. And piggybacking on the trust issue it’s going to be really important that that trust factor is there for them to receive what’s being said. So, I think all of that would come into play in terms of trying to encourage persons to become involved in something like that. So, its trust issue, how it’s presented, who presents it, and maybe where it’s presented.”
		Safety as a barrier to physical activity	“Cause a lot of people don’t like walking by theyself. Like me, I exercise by myself sometimes and it doesn’t bother me but people be telling me sometimes, ‘You shouldn’t be out here this early in the morning.’ But the gym that’s right close to me, they don’t open until ten o’clock in the morning.”<br><br>“Like walking, you don’t like to walk alone because it’s not safe nowadays.”
		Insufficient data plan or memory	“I think to make people aware that if you have limited data this is a possibility. So, I wasn’t aware of that in the beginning, I mean it didn’t matter to me but if I had known that and I had a limited plan then.”<br><br>[In response to "... any concerns about data plans do you think

			would come up with members of the community? Like taking too much data on their phone plans?"] “They probably would be.”
	**Perceived Susceptibility**		“Within our African-American community I think it is very important because statistics show, you know what they show, that you know we are a heavier population.”<br><br>“Piggybacking on what somebody said about the photos and African-American women because we’re like last in everything [health outcomes] because we don’t do what we supposed to do, some of us.”
	**Cues to action**		“The alarms and the reminders those were really helpful...Cause I would I realize I would sit there for three and four hours at a time, just doing work and not moving. So, those reminders were very helpful. I liked that.”
		Push notification messaging	“So, educating persons about the benefits of exercising and letting them know you know just like some of the messages say, ‘You don’t have to go to the gym. You don’t have to do two hours a day, you could break it up you know ten minutes.’ I mean getting that type of messaging out I think would be really helpful because I think if you didn’t grow up with it then we got to educate them and like some of the messages says, ‘It’s never too late.’ So, getting the message out to whatever the age population is I think is, would be a critical piece.”<br><br>“The one that says that, ‘Loving yourself means that you’re willing to take care of yourself. Keep that in mind your body is the only place, your body is your temple.’ And you know so those things are important and I know working with wellness centers and different things, they would be into this. That Vibrent app would be very good for them.”<br><br>“I would agree with that too because a lot of [the messages]

			talked about hair you know you know when we're exercising cause I would get that all the time, 'I don't exercise, I don't sweat because I don't want to sweat my hair out.' But I got a whole lot of messages that kind of referenced that hair."<br><br>"The messages were really good. They were very positive and they were really motivating and like [name redacted] said that, 'Don't quit. You know if you fell off the wagon today you know start again tomorrow.' So, I thought the messages were really good and very motivating."
		Expanding the definition of "exercise"	"I think for some people maybe would the app, maybe they don't have access to a gym but showing them what they can do even within they own home. Like you can't, maybe you can't go outside and walk, but you can do walk a mile in your home just walking or running in place. And you could do 10,000 steps doing that. Exercises that you could do in place of going to a gym."<br><br>"Yeah but you was saying for the ones who can't get out and go to a gym or whatever. Yard work definitely, doing laundry, going up and down your stairs which is another. And just walking back and forth in your house."
	**Self-efficacy**		[Speaking about the educational module that demonstrated stretching exercises] "And then when you look at the lady doing hers you're like, 'Oh I can do that, minus that one.' You know what I'm saying."<br><br>"So, I like that that it'll motivate me because I set my goal and most times I met my goal or went over the goal. But I went in there and set the goal that I want to do today or what I thought was manageable for me."<br><br>"One of mine was 'Move ten minutes longer, another day

			stronger.' I was like, 'Yeah, yeah I can do that! Ten minutes, yeah!' You know when the numbers is low it's like, 'Oh yeah, I can do that. I can do that.'"
**Emergent themes**			
	**Technical difficulties**		"I think my biggest thing is to make sure people can access the module. Because if people can't access it, you're talking about all this, I never could get into 'em. So, I don't know how helpful, it wasn't really helpful to me because I couldn't access it."<br><br>"And some days I didn't get [the daily self-assessment]. It didn't come up and then I'd have to do four for the days that I missed. Four would come up! It'd skip weeks, a week, and then I'd get four."<br><br>"Same thing, after I watched the modules it shut down. I didn't log myself out, I kept the whole thing open.…Then I finally found the password, you know, the email and the password in the folder and I kept using that over and over again, but it wouldn't let me [log in]."
		Check-ins or IT support	"I may not call…cause I don't think I ever called, but making sure that I know how to do it and I can do it or check in with me. Send me a text, 'How's it going? Are you having any issues?'"
	**Generational differences**		"But I do know that those of us who grew up with Physical Education in D.C. public schools, we're used to sort of even though it was back then, they don't do that now. So, they don't have even a jump start or somewhere to start from. So, it could make sense. Cause it was mandatory for those of us who grew up in the city, you had to. If you're not getting that from a younger age, you know, I don't know. I see the young men on the basketball courts and stuff but I rarely see...Like me for example those of us who rode bikes as a kid, I don't see young girls, I'm talking teenagers riding bikes. I rode my bike up

			until I started to drive. So, it's a population of young women right now that I see walking around that have no physical activity."<br><br>"I'm not going to say they're too busy, I think it's something that if you didn't grow up active, you not going to carry that over to adulthood."<br><br>"You know we do like to dance. So, that that's how, you know, a lot of us that still hand dance, or line dance, or whatever, I think something like that rather than, you know, just exercise might... Because they the young people do like music. So, something I don't know how you would do it but incorporate that into for younger people."
	**Relationship with community**		
		Connection to fellow participants	"Also, the group itself has been encouraging, so I know that after today we won't have the [group] so are we going to like meet once or twice a year or something to catch up, or is that something you all might want to do you know?"<br><br>"I also think that, you were talking about you're competitive I'm like, can we get a group going where we can you know compete against one another even after today? If I'm in a group but you know you can start several others. So, if we could get a group going for those who want to compete with one another it would help…It's not mandatory. It's for those who'd like to. It will definitely you know keep you motivated. (P: That sounds like a lot of fun.) And it's a lot of fun (P: It is, it is.) and it's a lot of fun to get those little messages like, 'Mmhmm okay, [name redacted] is only a 100 steps ahead of you.' And I'm like, 'Okay, well get up and walk.' So, it's a lot of it's a lot of fun, and I think when activities that you engage

			in, I think when they're fun as well, I think you're more inclined to stick with it and regardless of how much activity you engage in, it's a benefit."
		Connection to the research team	"Making sure that I can do it, before I go home because when I go home…I may not call…cause I don't think I ever called, but making sure that I know how to do it and I can do it or check in with me. Send me a text, 'How's it going? Are you having any issues?'"<br><br>"Good thing about it was if we needed y'all, y'all [were] available [to help during the study period]."<br><br>"I really would recommend a follow up. Because sometimes we start this, it's only three weeks and that's a short study you know but then how are these same people doing maybe six months down the road? You know as a follow up study so that then you can report back well what didn't work, was it a good introduction to have, how many are keeping up with it?"
	**Social support**		"Well, you know I said senior citizens because most of us we might go to a center and if we all have FitBits on it'll open up a conversation. (P: Mmhmm/P: It does) We'll start to do things."<br><br>"I know that when I started exercising I met [name redacted] and if I didn't show up for a few days [name redacted] was going to call me and say, 'Well, what's up with you? Are you okay?' So, it's sort of a checking…Yeah, accountability partner."<br><br>[Speaking about the content of the messages] "Because it made me feel as a part of the community of women that are trying to improve our health."
	**Preferred features**		"I like it when they have this little red thing right here where

		they jump up. Because it makes you move. […] You got to move! You got what, 130 more steps to make your 250. [Speaking about the hourly Fitbit reminders to take 250 steps]<br><br>"I liked when it captured how much sleep you had. (P: We are on the same page.) I was like, 'Oh my God I only slept three hours.' So, and I said, 'The next day get in the bed a little bit early.' Because I'm wanting to do stuff around the house until I would drop dead tired and then want to get in the bed. So, it's like 'Okay no stop at a certain time and then get in the bed so you could get some more sleep.' So I really liked that part."<br><br>"Well, I did like the video also. And also, with mines I could go back to it anytime I wanted to review it. [...] I could just go back to it and watch it if I wanted encouragement or something like that."<br><br>[Speaking about the wall of messages] "That's [the wall] helpful because sometimes you need to see these things over and over you know repetition helps you keep things in your head. It'll motivate you."
